# A Multidisciplinary Consensus‐Building Exercise to Define and Prioritize Topics in Supportive Care of Children With Cancer at a Global Level

**DOI:** 10.1002/cam4.70377

**Published:** 2024-11-05

**Authors:** Elizabeth Sniderman, Tea Reljic, Manoo Bhakta, Miguel Bonilla, Julie Clyce, Jessica Farmer, Monica Key, Sergio Licona, Jennifer L. Pauley, Alondra Torres‐Gonzalez, Michael Sullivan, Nickhill Bhakta, Ambuj Kumar, Sheena Mukkada

**Affiliations:** ^1^ Department of Global Pediatric Medicine St. Jude Children's Research Hospital Memphis Tennessee USA; ^2^ Research Methodology and Biostatistics Core, College of Medicine Office of Research University of South Florida Tampa Florida USA; ^3^ Biology Department University of Puerto Rico‐Arecibo Arecibo Puerto Rico; ^4^ Department of Paediatrics Children's Cancer Centre, Royal Children's Hospital Melbourne, ACT Australia

**Keywords:** childhood cancer, global oncology, pediatric oncology, supportive care

## Abstract

**Introduction:**

Optimal outcomes during childhood cancer treatment require effective management of toxicities, often called supportive care. A lack of agreement on what comprises supportive care limits the development and provision of comprehensive guidance (for this work, we have defined supportive care as any disease‐ or treatment‐related condition experienced by children with cancer, excluding psychosocial conditions, palliative care, survivorship, or procedural topics). To address this gap, we conducted a consensus‐building exercise among global experts to define and prioritize topics for supportive care.

**Methods:**

Two rounds of brainstorming and prioritization exercises were conducted. A multidisciplinary panel nominated by professional societies and cooperative groups was formed to ensure geographic and resource representation using snowball sampling. An internal expert panel generated an initial list of supportive care topics. In round one, the multidisciplinary panel reviewed the initial list and recommended additional topics, followed by prioritization in round two using a seven‐point Likert scale. Results were summarized using descriptive statistics.

**Results:**

The multidisciplinary panel consisted of 57 members representing 32 countries. The initial list included 46 topics; 161 additional topics were suggested. After removing duplicates and out‐of‐scope additions, the final list contained 62 topics. Febrile neutropenia, sepsis, bloodstream infections, and pain were ranked highest priority. Mortality, morbidity, and frequency of the event were identified as the most important factors influencing prioritization.

**Conclusion:**

Through a multidisciplinary and globally representative process, we identified core supportive care topics and factors influencing their prioritization for childhood cancer. Outputs from this work will inform efforts to generate resource‐adapted recommendations for a global audience. This supports ongoing WHO CureAll work to develop a health systems‐level policy brief of supportive care requirements in the management of children with cancer.

## Graphical abstract

High‐quality supportive care is critical to improve outcomes for childhood cancer and mitigate the treatment‐related toxicity and mortality of increasingly potent treatment regimens. Sixty‐two supportive care topics were identified and rank‐ordered by a global, multidisciplinary panel of pediatric oncology clinicians to guide development of resource‐adapted clinical practice recommendations; morbidity and mortality were identified as the key criteria for prioritizing topics.

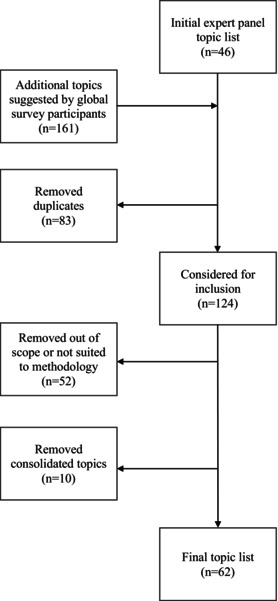

## 1 Introduction

High quality, evidence‐based care to manage the expected toxicities of treatment is essential to improve outcomes for childhood cancer in all settings [1, 2, 3, 4, 5, 6]. In part due to advances in infection management, nutrition, and other interventions typically labeled as “supportive care,” 5‐year overall survival in high‐income countries (HICs) has surpassed 85% [6, 7, 8]. While improvements in survival outcomes are also attributed to increased treatment intensity for certain cancer subtypes, these treatment plans must be accompanied by high‐quality supportive care guidance and resources to prevent excess toxicity and treatment‐related mortality, especially in limited‐resource settings [1, 4, 9].

Despite the importance of supportive care interventions, there is a lack of normative consensus on how to define what constitutes supportive care. The Multinational Association of Supportive Care in Cancer (MASCC) defines supportive care as “the prevention and management of the adverse effects of cancer and its treatment across the cancer continuum” [10] without consensus on what elements are included in this broad definition. As supportive care needs vary based on individual and treatment setting related factors, such as the type of cancer‐directed therapy administered, available human and material resources, and local preferences, no uniform index of supportive care topics exists. A consensus‐exercise from the Netherlands [11] used the Delphi approach to identify local priorities for development of national supportive care clinical practice guidelines. Independently, the SIOP PODC (International Society of Pediatric Oncology [SIOP] Pediatric Oncology in Developing Countries, now SIOP Global Health Network) published a limited set of supportive care recommendations directed at low resource settings, but these were not informed by a formal needs assessment [4]. To date, there has been no comprehensive attempt to define the scope and significance of supportive care that spans all health delivery contexts, considering both geographic and resource heterogeneity, and including inpatient or outpatient medical, surgical, and radiotherapy treatment settings [11, 12]. This leads to divergent, time‐intensive efforts by individual disciplines, centers, countries, or regions to develop guidelines of varying methodologic quality. To address this gap, the ARIA (Adapted Resource and Implementation Application) Guide is being developed by global stakeholders in pediatric oncology (SIOP, St. Jude Children's Research Hospital, Childhood Cancer International, Pediatric Radiation Oncology Society, and the International Society of Pediatric Surgical Oncology) and incorporates both disease‐based and supportive care treatment guidelines, along with palliative care and childhood cancer survivorship recommendations. We conducted a multidisciplinary, consensus‐building exercise consisting of a globally representative panel to identify and prioritize supportive care topics. This work will inform future development resource‐adapted supportive care clinical practice recommendations.

## 2 Methods

We used a cross sectional design. A ranking exercise was developed and administered using Qualtrics survey software via email in two rounds. This work was reviewed by the Institutional Review Board at St. Jude Children's Research Hospital and determined to be exempt as secondary research. The Institutional Review Board at St. Jude Children's Research Hospital waived the need for informed consent.

### 2.1 Initial Topic List and Internal Expert Panel

An internal expert panel was comprised of five pediatric oncologists with varying years of clinical experience, one pediatric oncology nurse practitioner, one pharmacist, and one pediatric infectious diseases physician, all of whom are a part of the core ARIA Guide Supportive Care team. The scope of topics was defined to include disease‐ or treatment‐related conditions experienced during childhood cancer care. Psychosocial, palliative care, survivorship/long‐term side effects, and procedure‐based (e.g., central line care) topics were considered as out of scope for this exercise, as they are addressed in other areas of the ARIA Guide. Initial topics were extracted from three sources: (1) needs identified during development of the ARIA Guide disease‐based guidelines; (2) topics selected through a survey to define high priority adverse events for global data capture [13]; and (3) additional input from the multidisciplinary content expert panel. This produced an initial list of 46 topics.

### 2.2 Setting up a Multidisciplinary Expert Panel

We used snowball sampling to establish the global multidisciplinary expert panel. As the goal of the exercise was to identify and prioritize supportive care topics for childhood cancer care, we contacted pediatric oncology professional societies and cooperative groups to nominate participants for the expert panel. These groups were asked to contribute representatives from the spectrum of disciplines that provide supportive care to children with cancer. Our sampling strategy aimed to diversify country representation by World Bank income classification and World Health Organization (WHO) region (Appendix 1). The same individuals were invited to participate in both rounds of the exercise.

### 2.3 Endorsement of Guiding Principles for Supportive Care in Childhood Cancer

Prior to defining and prioritizing topics, the multidisciplinary expert panelists were asked their level of agreement with nine guiding principles. The purpose of these statements, developed by the ARIA Guide Supportive Care team, was to evaluate whether panelists had a shared understanding of the scope and importance of creating evidence‐based supportive care guidance. In the first survey round, participants rated their agreement with each statement on a 9‐point Likert scale (1 = strongly disagree, 9 = strongly agree). Individual agreement with each principle was defined a priori as any score ≥ 7; free‐text comments were required on any statements rated < 7 (Appendix 2).

### 2.4 Topic Selection and Prioritization

The prioritization task was performed in two rounds. Responses to the first round were collected from October 26 to November 11, 2022. Following analysis of the results, a second round was conducted, with responses collected from December 9 to December 27, 2022.

In the first round, the global multidisciplinary panel members received the initial list of supportive care topics for review with the invitation to insert any additional topics they deemed important in the supportive care of children during cancer treatment. The panel members were then asked to rank the relative importance of the seven criteria they used to prioritize supportive care topics (“prioritization criteria”) and suggest any additional prioritization criteria. In all rounds, the participants were provided with an option to provide additional qualitative comments.

Following the first round, the internal content experts reviewed the suggested additional topics and prioritization criteria for inclusion. In the second round of the survey, participants prioritized final topics using a 7‐point Likert scale (1 = very low priority, 7 = very high priority). Participants were then asked to rank the prioritization criteria they had considered in the previous step of the survey.

### 2.5 Data Analysis

All complete responses were included for analysis. The results were summarized descriptively using measures of central tendency (mean), variance (SD or range), and rates/percent.

## 3 Results

### 3.1 Characteristics of the Global Multidisciplinary Panel and Response Rate

Of the 91 nominees, 50 agreed to participate in round 1 of the exercise. Of these, 5 did not complete the questionnaire, resulting in a final count of 45 individual responses to round 1 (55% response rate; 49.5% completion rate). In round 2, 53 individuals agreed to participate, and 46 completed the survey (58% response rate; 50.5% completion rate). Of 57 individual respondents to either round, 22 responded to only one round, while 35 responded to both. Participants represented 32 individual countries. Respondents from low‐ or middle‐income countries made up 68% of total respondents (39/57), representing 69% (22/32) individual countries and all WHO regions (Appendix 3). Participant demographics are presented in Table 1.

**TABLE 1:** Respondent demographics.

	LMIC	UMIC	HIC	Total
Gender				57
Male	11	6	11	28
Female	9	14	6	29
WHO Region				57
AFR	4	2	0	6
AMR	0	14	9	23
EMR	7	3	1	11
EUR	0	1	6	7
SEAR	7	0	0	7
WPR	0	2	1	3
Discipline				57
Pediatric Oncologist	13	12	8	33
Radiation Oncologist	2	3	4	9
Surgeon	0	3	3	6
Nursing	1	1	1	3
Pharmacy	3	1	1	5
Non‐profit professional	1	0	0	1

Abbreviations: AFR, African Region; AMR, Region of the Americas; EMR, Eastern Mediterranean Region; EUR, European Region; HIC, high income countries; LMIC, low‐ or middle‐income countries; SEAR, South‐East Asia Region; UMIC, upper middle‐income countries; WPR, Western Pacific Region. Country income classifications are according to the World Bank (2023) [27]. WHO regions are according to the World Health Organization (2023) [28].

### 3.2 Guiding Principles

All nine guiding principles regarding evidence‐based guidance for supportive care of children with cancer were rated as “agreed.” Table 2 presents the mean rate of agreement with the guiding principles and percentage of participants rating ≥ 7.

**TABLE 2:**
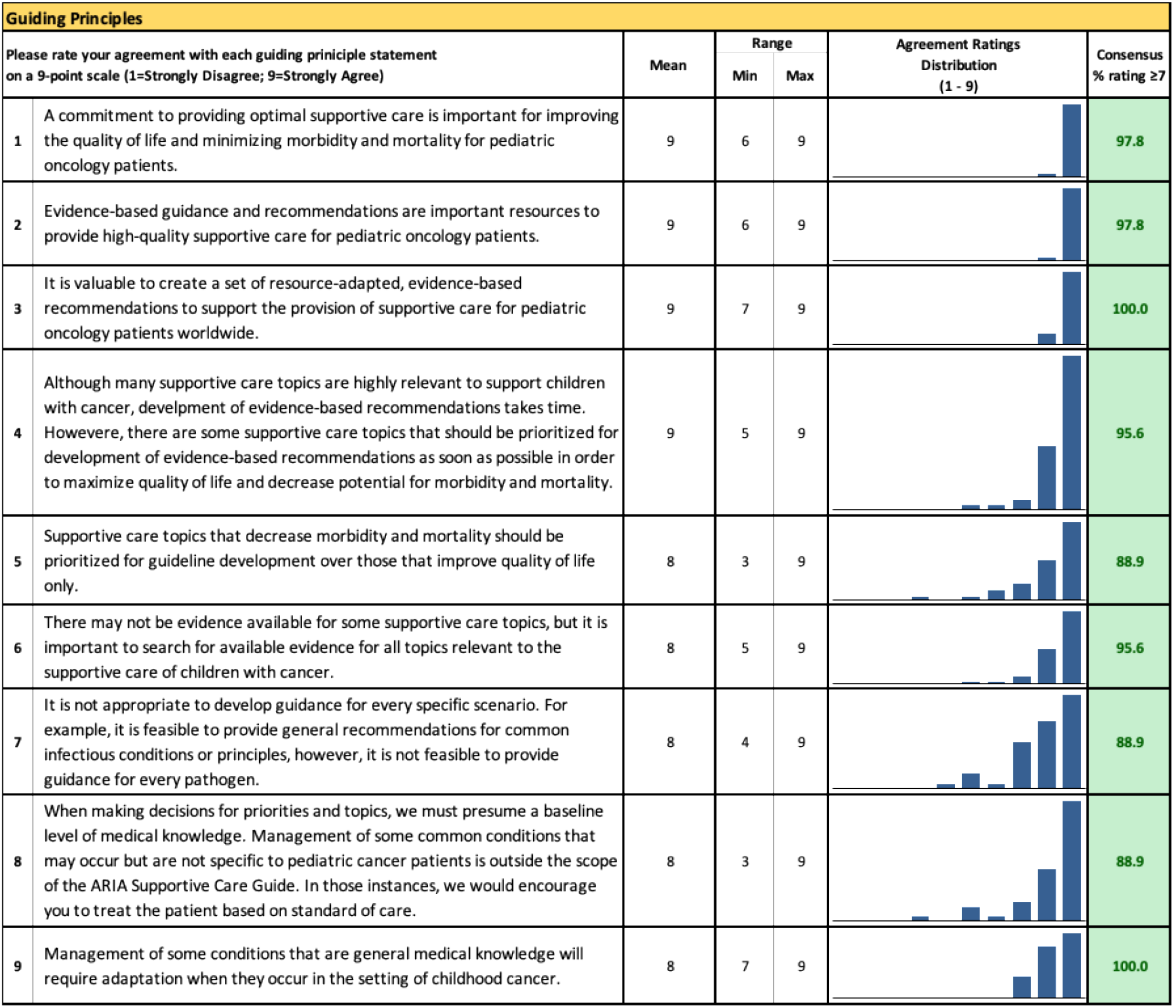
Rating of guiding principles for the global childhood cancer supportive care prioritization exercise (Round 1).

### 3.3 Topic List and Priority Assignment

**FIGURE 1:**
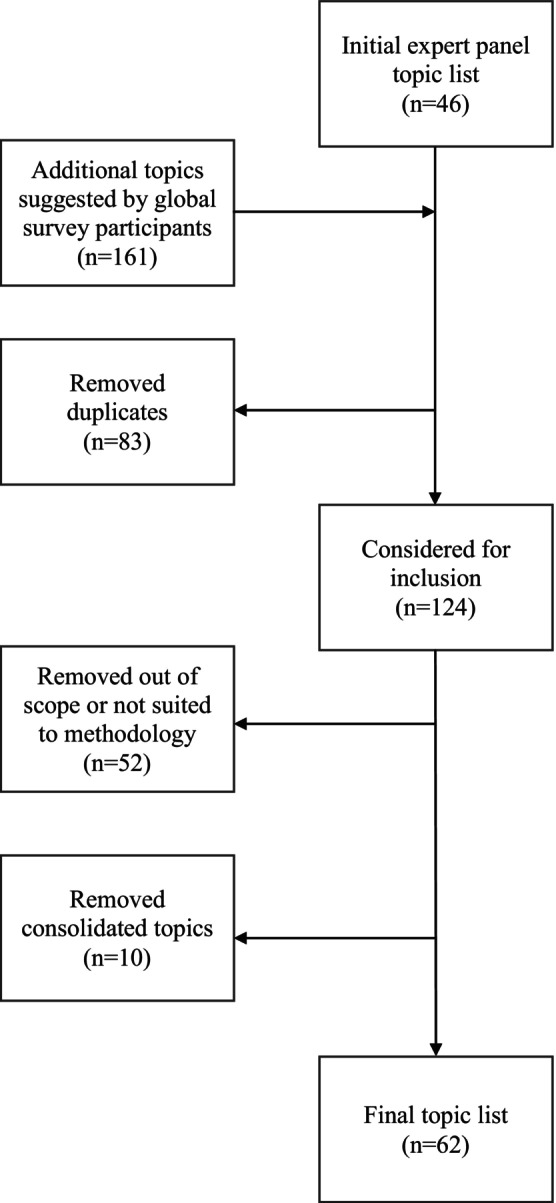
Flowchart of creation of the final topic list.

As illustrated in Figure 1, the original topic list consisted of 46 topics. During round 1, respondents approved the original topics and suggested an additional 161 items. Eighty three items were removed as duplicates of previously included supportive care topics (*n* = 43) or duplicate suggestions (*n* = 40). Of the 124 items, 52 were considered outside the scope of this exercise, as determined by the internal content experts. Additionally, due to overlap among topics, 10 items were removed to combine topics (e.g., 13 topics were combined into the umbrella topic of “radiotherapy complications”, and “urinary retention [due to obstruction]” was combined with an original topic [“spinal cord compression”]). This resulted in a total of 62 topics (Figure 1).

Final prioritization for the 62 topics is shown in Table 3, along with mean scores. All topic means ranged from 4.5 to 6.7 on a 7‐point Likert scale, with 7 denoting highest priority. The top five topics, according to their mean, were fever and neutropenia (mean = 6.7), sepsis (mean = 6.6), culture‐positive bloodstream infections (mean = 6.3), pain (mean = 6.3), and mediastinal mass (mean = 6.3).

**TABLE 3:** Final supportive care topic list in priority order (Round 2).

Rank	Supportive care topic	Mean	SD
1	Fever and neutropenia	6.7	0.7
2	Sepsis and septic shock	6.6	0.8
3	Culture‐positive bloodstream infections	6.3	0.9
4	Pain	6.3	0.9
5	Mediastinal mass	6.3	1.1
6	Tumor lysis syndrome	6.2	1.3
7	Catheter‐related infection	6.2	0.9
8	Nutrition	6.1	1.0
9	Methotrexate‐related toxicities	5.9	1.1
10	Disseminated intravascular coagulation	5.9	1.1
11	Mucositis	5.9	1.3
12	Increased intracranial pressure	5.9	1.2
13	Fungal prophylaxis	5.9	1.2
14	Chemotherapy‐induced nausea and vomiting	5.8	1.1
15	Seizure	5.8	1.2
16	Infusion site extravasation	5.8	1.2
17	Thromboembolic events	5.7	0.9
18	Spinal cord compression	5.7	1.5
19	Bacterial prophylaxis	5.7	1.3
20	Typhlitis	5.7	1.0
21	Radiation therapy complications	5.6	1.0
22	Candidiasis	5.6	1.0
23	Hyperleukocytosis	5.6	1.5
24	Oral care	5.5	1.2
25	Pancreatitis	5.5	1.2
26	Blood product transfusion indications	5.5	1.4
27	Respiratory viral infections	5.5	1.2
28	Thrombocytopenia	5.5	1.4
29	Drug‐induced nephrotoxicity	5.4	1.1
30	Anemia	5.4	1.2
31	Anaphylaxis	5.4	1.4
32	Hemorrhagic cystitis	5.4	1.2
33	Posterior reversible encephalopathy syndrome	5.4	1.2
34	Drug‐induced encephalopathy	5.4	1.4
35	Hypertension	5.4	1.4
36	*Pneumocystis jirovecii* pneumonia	5.3	1.2
37	*Clostridium difficile* infection	5.3	1.2
38	Ototoxicity	5.3	1.2
39	Drug‐induced liver injury	5.2	1.2
40	Stroke	5.2	1.4
41	Varicella zoster virus	5.2	1.1
42	Posterior fossa syndrome	5.2	1.3
43	Syndrome of inappropriate secretion of antidiuretic hormone	5.2	1.3
44	Cardiomyopathy	5.2	1.1
45	Chemotherapy‐induced peripheral neuropathy	5.2	1.2
46	Pleural effusion	5.2	1.2
47	Chemotherapy‐associated diarrhea	5.1	1.2
48	Sinusoidal obstruction syndrome	5.1	1.2
49	Immunizations	5.1	1.3
50	Pericardial effusion	5.1	1.2
51	Wound complication	5.0	1.3
52	Osteonecrosis	5.0	1.1
53	Drug‐induced hyperglycemia	4.9	1.3
54	Indications for stress‐dose hormone	4.9	1.3
55	Herpes simplex virus	4.8	1.3
56	Fertility preservation	4.8	1.5
57	Constipation	4.7	1.4
58	Skin complications	4.7	1.3
59	Tuberculosis	4.6	1.5
60	Parasitic infections	4.6	1.6
61	HIV infection	4.5	1.5
62	Hormone replacement therapy	4.5	1.1

### 3.4 Prioritization Criteria Ranking

In round 1, respondents recommended 11 additional prioritization criteria that could be considered for creation of evidence‐based recommendations for supportive care topics. The study team reviewed these recommendations and added two prioritization criteria that were not encompassed by any of the original seven. These were importance to patients and families and cost/benefit of management of the condition.

In both rounds, risk of death (mortality) due to condition, risk of serious or poor adverse events (morbidity) due to condition, and frequency (how commonly this occurs) were rated as the most important prioritization criteria. Overall, prioritization criteria were ranked in the same order prior to the prioritization exercise (in round 1) and post hoc, after prioritizing topics (in round 2), apart from ability to have an impact on disease outcomes/survival and ability to prevent the condition, which reversed ranking order (third and fourth) in the second round. Ordering of prioritization criteria with mean scores is presented in Table 4.

**TABLE 4:** Ranking of prioritization criteria.

Rank (Round 1)	Rank (Round 2)	Prioritization criteria	Mean	SD
1	1	Risk of death (mortality) due to condition	2.7	1.9
2	2	Risk of serious or poor adverse events (morbidity) due to condition	2.9	1.1
3	3	Frequency (how commonly this occurs)	3.2	2.3
5	4	Ability to have an impact on disease outcomes/survival	4.3	1.5
4	5	Impact on the patient's quality of life	4.7	2.3
6	6	Ability to prevent the condition	5.4	1.7
7	7	Availability of resources needed to prevent or manage the condition	6.3	1.7
*n*/a	8	Importance to patients and families^a^	7.4	1.7
*n*/a	9	Cost/benefit of management of the condition^a^	8.2	1.6

^a^ New prioritization criteria added by participants during Round 1.

## 4 Discussion

We successfully identified and prioritized topics for development of resource‐adapted supportive care guidance using a global multidisciplinary panel of experts. The WHO Global Initiative for Childhood Cancer aims to achieve at least 60% survival and reduce suffering for all children with cancer by 2030 [6]. The CureAll framework operationalizes this goal, advising stakeholders to develop national standards of care for childhood cancer management along the patient pathway “and consider essential supportive care services and treatment‐related toxicities” [6]. By identifying these priority topics for policymakers and health systems administrators, our work can support development of policies and practice briefs. This may prevent investment in therapy for curative intent without adequate attention to supportive care needs as perceived by global providers. Our results will inform the development of clinical practice recommendations in future global efforts.

To date, the term supportive care remains broad. The definition used by MASCC [10] defines supportive care but does not provide a comprehensive list of what topics must be included for guidance, nor which should be prioritized. Our global, multidisciplinary process built on the list from the SIOP PODC recommendations, which included seven individual topics, in addition to statements recommending the provision of palliative care, psychosocial support, and practical suggestions for infection prevention, nursing care, and chemotherapy delivery [3], and the topics identified as priorities through the Delphi process conducted by Loeffen et al. among childhood cancer providers in the Netherlands (infection, sepsis, febrile neutropenia, pain, and nausea/vomiting) [11]. Additional priority topics identified by the global multidisciplinary panel included mediastinal mass, tumor lysis syndrome, and disseminated intravascular coagulation.

Several topics identified as priorities in our global survey highlight the differences in disease‐ and treatment‐related morbidity and mortality in lower‐resourced settings. Late presentation and higher burden of disease are common in low‐ or middle‐income countries due to limited awareness of early signs of cancer, barriers in accessing care, and scarce resources; this may increase the risk for bulky mediastinal mass, tumor lysis syndrome, and hyperleukocytosis [14, 15]. These topics were ranked 5th, 6th, and 23rd priority, respectively, by our participants but were not included in the list from Loeffen et al. [11] The inclusion of methotrexate‐related toxicity on our list may reflect the lack of drug‐level monitoring capacity in low‐ or middle‐income countries [5]. Nutrition was also ranked higher by global participants, potentially reflecting the high burden of malnutrition in childhood cancer patients globally [5, 6, 15, 16]. These differences illustrate our major contribution to the literature, which is the global perspective on the priority of supportive care needs during treatment of childhood cancer.

In the survey by Loeffen et al., respondents were asked to score each topic based on prevalence, severity, and whether adequate treatment options existed for the condition [11]. While we allowed our respondents to select priority topics without stipulating how they should prioritize, they voted that mortality, morbidity, and frequency of the adverse event or side effect of treatment were the key prioritization criteria driving decision‐making in both round 1 (before completing the ranking exercise) and round 2 (after completing the ranking exercise). These criteria have been previously described in work aimed at identifying and developing supportive care guidance in childhood cancer [4, 17]. Long‐term toxicities, such as those elucidated by Andres‐Jensen et al. in acute lymphoblastic leukemia, have a significant effect on survivors [18]. Our findings highlight that currently, many global respondents prioritized helping a child to successfully complete cancer therapy and survive acute toxicities. This exercise accordingly sets current priorities for guideline development.

As 90% of children with cancer live in low‐and‐middle‐income‐countries, it is critical to allow for representative sampling to define the agenda for work. Evidence‐based guidance to enhance supportive care is critical to improving outcomes for children undergoing cancer treatment in low‐ and middle‐income countries [1, 2, 3, 4, 5, 6, 10, 14, 15, 16, 19, 20, 21]. In a recent systematic review, the burden of treatment‐related mortality was found to be two times higher in lower‐middle‐income countries and three times higher in low‐income countries than in HICs, accounting for 30.9% of all childhood cancer deaths in low‐ or middle‐income countries [1]. Improved supportive care interventions for infections, fever and neutropenia, tumor lysis syndrome, nutrition, anemia, and thrombocytopenia led to a decrease in toxic deaths from 25.7% to 11.6% during B‐cell lymphoma treatment in Northern Africa over a 3 year period [2, 22]. Beyond mortality, adverse effects which can be mitigated with supportive care adversely affect patient quality of life. They also have the hidden cost of undermining the tolerability of current treatment regimens, which can cause patients and families to refuse or abandon treatment [5, 15, 21].

The principal strength of our work is our incorporation of stakeholders from a broad geographic reach. For this consensus‐building exercise, we attempted to recruit participants that would represent all World Bank country income classifications and WHO regions. The skewed representation of participants from high‐income and upper‐middle‐income countries is related to the overall shortage of healthcare workforce in lower‐resourced settings [23]. Additionally, because the survey was only available in English, we have lower representation in WHO regions where English is less commonly used for communication. Seven disciplines were invited to participate in the exercise; 60% of participants were pediatric oncologists. The under‐representation of other disciplines and lack of inclusion of sub‐specialties (e.g., infectious diseases or critical care experts) may be due to the composition of the pediatric oncology professional societies and cooperative groups that were approached to nominate participants; additionally, patients and families were not approached to participate in the exercise. This under‐representation may limit interpretation of the prioritization results. In particular, the paucity of nursing representation may have biased the results. Future work should focus on systematically eliciting nursing perspective on priorities along with increasing participation by additional disciplines.

A key limitation of our work is our scope; since our practical goal was to develop provider‐focused clinical practice recommendations using a standard methodology, we intentionally defined supportive care for this exercise as any disease‐ or treatment‐related condition experienced by children with cancer [11], excluding psychosocial conditions, palliative care, survivorship, or procedural topics. Our multidisciplinary panel elicited the provider's perspective on what they perceived as needs to provide optimal supportive care. Psychosocial aspects of care, hygiene, and physical activity were suggested by participants during round 1 and have been identified by patients and families as critical supportive care topics; however, they merit a dedicated exercise incorporating other stakeholders such as child life specialists, psychologists, psychiatrists, and patient and family representatives [24]. Similarly, palliative and survivorship care needs should also be defined by a different and broader stakeholder panel. The evidence base needed to create procedural instructions for procedural‐based topics, such as central venous line care and insertion of nasogastric tubes, is sufficiently different from that needed to create clinical practice recommendations for toxicities. Therefore, these topics were intentionally excluded from our list in favor of ensuring that the appropriate stakeholders and experts are included in prioritization and development of such topics for future inclusion in the ARIA Guide as an important element of high‐quality multidisciplinary care for children with cancer. By documenting our approach, we enable replication by others who want to use our method and help others understand how they can use our work as a foundation.

## 5 Conclusion

The results of this global consensus‐building exercise inform the agenda for supportive care in global pediatric oncology. While the importance of creation of evidence‐based clinical practice guidelines has been previously emphasized [10, 11, 12, 17, 25, 26], little attention has been paid to the adaptation for variously resourced settings, nor to the dissemination of guidelines in a pragmatic, accessible, and interactive manner. The ARIA Guide aims to address the global need for comprehensive, resource‐stratified childhood cancer management guidelines on a free, intuitive platform that includes implementation tools needed to support day‐to‐day care. Over the next several years, guidance for the supportive care topics identified will be developed based on a systematic methodology to identify, appraise, and adapt the existing clinical practice guidelines for variably resourced settings.

## Author Contributions

**Elizabeth Sniderman:** conceptualization (equal), data curation (equal), formal analysis (equal), investigation (equal), methodology (equal), project administration (equal), writing – original draft (lead). **Tea Reljic:** conceptualization (equal), formal analysis (equal), methodology (equal), project administration (supporting), software (equal), writing – review and editing (equal). **Manoo Bhakta:** methodology (supporting), writing – review and editing (equal). **Miguel Bonilla:** methodology (supporting), writing – review and editing (equal). **Julie Clyce:** conceptualization (supporting), project administration (supporting), writing – review and editing (equal). **Jessica Farmer:** project administration (supporting), writing – review and editing (equal). **Monica Key:** formal analysis (supporting), project administration (supporting), writing – review and editing (equal). **Sergio Licona:** conceptualization (supporting), methodology (supporting), project administration (supporting), writing – review and editing (equal). **Jennifer L. Pauley:** conceptualization (supporting), methodology (supporting), writing – review and editing (equal). **Alondra Torres‐Gonzalez:** conceptualization (supporting), methodology (supporting), project administration (supporting), writing – review and editing (equal). **Michael Sullivan:** supervision (equal), writing – review and editing (equal). **Nickhill Bhakta:** conceptualization (supporting), methodology (supporting), resources (lead), supervision (equal), visualization (equal), writing – original draft (supporting), writing – review and editing (equal). **Ambuj Kumar:** conceptualization (supporting), formal analysis (supporting), methodology (equal), supervision (equal), validation (equal), writing – review and editing (equal). **Sheena Mukkada:** conceptualization (equal), formal analysis (equal), investigation (equal), methodology (equal), project administration (supporting), supervision (lead), validation (equal), visualization (equal), writing – original draft (equal).

## Conflicts of Interest

The authors declare no conflicts of interest.

## Data Availability

The data sets that support the findings of this study are available from the corresponding authors on request.

## Appendix 1 Countries Contacted to Participate in Prioritization Exercise

### 1.1

**Table:**

	Country name
1	Argentina
2	Australia
3	Brazil
4	Cambodia
5	Cameroon
6	Canada
7	Chile
8	China
9	Costa Rica
10	Czech Republic
11	Denmark
12	Egypt
13	El Salvador
14	Germany
15	Guatemala
16	India
17	Iran
18	Iraq
19	Japan
20	Jordan
21	Kenya
22	Lebanon
23	Malaysia
24	Mexico
25	Morocco
26	Netherlands
27	Nicaragua
28	Pakistan
29	Peru
30	Portugal
31	Qatar
32	Saudi Arabia
33	Singapore
34	South Africa
35	Tanzania
36	Tunisia
37	Turkey
38	Uganda
39	United Kingdom
40	United States of America
41	Venezuela
42	Vietnam
43	Zambia

## Appendix 2 Free‐Text Comments on Guiding Principles for the Global Childhood Cancer Supportive Care Prioritization Exercise (Round 2)

### 2.1

**Table:**

	Statement	Comment (s)
1	A commitment to providing optimal supportive care is important for improving the quality of life and minimizing morbidity and mortality for pediatric oncology patients.	“The statement needs to emphasize primarily on decreasing ‘morbidity and mortality,’ especially treatment/disease related morbidity. The ‘quality of life’ phrase in my opinion should come in latter part of the statement. Since quality of life is so intricately related to morbidity, I am not so convinced that the phrase ‘quality of life’ needs to be included in a supportive care context.”
2	Evidence‐based guidance and recommendations are important resources to provide high‐quality supportive care for pediatric oncology patients.	“Evidence base is essentially based on data from HIC. I am not sure we can really talk about evidence base when we talk about LMIC.”
3	It is valuable to create a set of resource‐adapted, evidence‐based recommendations to support the provision of supportive care for pediatric oncology patients worldwide.	(None)
4	Although many supportive care topics are highly relevant to support children with cancer, development of evidence‐based recommendations takes time. However, there are some supportive care topics that should be prioritized for development of evidence‐based recommendations as soon as possible in order to maximize quality of life and decrease potential for morbidity and mortality.	“I would first prioritize supportive care that decreases potential for morbidity and mortality. The QoL is important, however, much less than supportive care that could be life saving.”
5	Supportive care topics that decrease morbidity and mortality should be prioritized for guideline development over those that improve quality of life only.	“I think it is important to do both although the immediate need for many places first maybe morbidity and mortality guidelines.” “In some settings, it is only possible to provide quality of life only. Sometimes because of lack of resources to provide curative treatment or because of the type of disease.” “Quality of life should have the same weight as morbidity. They go hand in hand.” “We need to adopt medicine and conduct that treat the patient in all his physical, psychic, religious, and holistic needs.”
6	There may not be evidence available for some supportive care topics, but it is important to search for available evidence for all topics relevant to the supportive care of children with cancer.	“I agree with the principle of searching for evidence, but if this requires a lot of resources to do this for ‘all topics’, these resources might be better deployed to help with generating new evidence rather than searching for poor quality evidence where it barely exists.” “We must respect all the cultural and religious aspects of the patients sometimes not pointed out in the literature.”
7	It is not appropriate to develop guidance for every specific scenario. For example, it is feasible to provide general recommendations for common infectious conditions or principles; however, it is not feasible to provide guidance for every pathogen.	“Common infectious conditions are not the same in all countries.” “I believe some items with high impact on patient outcomes need to be addressed specifically. For example, in relation to infectious disease, addressing the management of infections with multi‐drug resistant organisms is vital and each one should be talked separately.” “In a low‐income country, it could be difficult some drugs, for example. An option could be thought of together.” “It may be more difficult to work with all the causes, but it must be tried; the more information we can give is of greater value for the treatment.” “There are several specific situations where certain pathologies will behave in a unique pattern and therefore even though general principles are the base of a guide, certain situations should be stressed in order to avoid pitfalls in management.”
8	When making decisions for priorities and topics, we must presume a baseline level of medical knowledge. Management of some common conditions that may occur but are not specific to pediatric cancer patients is outside the scope of the ARIA Supportive Care Guide. In those instances, we would encourage you to treat the patient based on standard of care.	“I agree that this statement might be true in most of the cases. However, in centers where children with cancer are treated in non‐oncology centers (e.g., in general pediatric wards or under adult oncology), then it might be better to make sure management is adequate for these patients and would not put them at risk of complications. In other words, children in such situations might not be regarded as high or higher risk.” “We need to agree on ‘not specific to pediatric cancer patients.’ As an example, endocrine issues that can be life threatening in some patients (craniopharyngioma, intracranial germ cell tumors) are not specific to pediatric cancer patients. However, they are critical in my opinion.” “We should work with standard information, but if there is a need to expand this information with more specific and detailed knowledge, I am a supporter of the more information with clear content and in easy access language, and reading together the better to conduct the treatment.” “While some conditions may be not specific for cancer patients, the pediatric cancer patient in itself is a very unique condition of health and should be treated as one.”
9	Management of some conditions that are general medical knowledge will require adaptation when they occur in the setting of childhood cancer.	(None)

Abbreviations: HIC, high‐income countries; LMIC, low‐ or middle‐income countries; QoL, quality of life.

## Appendix 3 Global Participation in Prioritization Exercise Based on Frequency of Responses Per Country (Total Respondents: 57)

### 3.1

**Table:**

Guiding principles							Response values									
Please rate your agreement with each guiding priniciple statement on a 9‐point scale (1 = Strongly Disagree; 9 = Strongly Agree)		Mean	Range		Agreement *R* = ratings distributio*n* (1–9)	Consensus % rating ≥ 7	1	2	3	4	5	6	7	8	9	*N*
			Min	Max												
**1**	A commitment to providing optimal supportive care is important for improving the quality of life and minimizing morbidity and mortality for pediatric oncology patients.	9	6	9		97.8	0	0	0	0	0	0	1	0	2	42
**2**	Evidence‐based guidance and recommendations are important resources to provide high‐quality supportive care for pediatric oncology patients.	9	6	9		97.8	0	0	0	0	0	0	1	0	2	42
**3**	It is valuable to create a set of resource‐adapted, evidence‐based recommendations to support the provision of supportive care for pediatric oncology patients worldwide.	9	7	9		100.0	0	0	0	0	0	0	0	1	6	38
**4**	Although many supportive care topics are highly relevant to support children with cancer, development of evidence‐based recommendations takes time. Howevere, there are some supportive care topics that should be prioritized for development of evidence‐based recommendations as soon as possible in order to maximize quality of life and decrease potential for morbidity and mortality.	9	5	9		95.6	0	0	0	0	0	1	1	2	12	29
**5**	Supportive care topics that decrease morbidity and mortality should be prioritized for guideline development over those that improve quality of life only.	8	3	9		88.9	0	0	0	1	0	1	3	5	12	23
**6**	There may not be evidence available for some supportive care topics, but it is important to search for available evidence for all topics relevant to the supportive care of children with cancer.	8	5	9		95.6	0	0	0	0	0	1	1	3	13	27
**7**	It is not appropriate to develop guidance for every specific scenario. For example, it is feasible to provide general recommendations for common infectious conditions or principles; however, it is not feasible to provide guidance for every pathogen.	8	4	9		88.9	0	0	0	0	1	3	1	9	13	18
**8**	When making decisions for priorities and topics, we must presume a baseline level of medical knowledge. Management of some common conditions that may occur but are not specific to pediatric cancer patients is outside the scope of the ARIA Supportive Care Guide. In those instances, we would encourage you to treat the patient based on standard of care.	8	3	9		88.9	0	0	0	1	0	3	1	4	11	25
**9**	Management of some conditions that are general medical knowledge will require adaptation when they occur in the setting of childhood cancer.	8	7	9		100.0	0	0	0	0	0	0	0	7	17	21

## References

[1] B. S. Ehrlich , M. J. McNeil , L. T. D. Pham , et al., “Treatment‐Related Mortality in Children With Cancer in Low‐Income and Middle‐Income Countries: A Systematic Review and Meta‐Analysis,” Lancet Oncology 24, no. 9 (2023): 967–977, 10.1016/S1470-2045(23)00318-2. 37517410 PMC10812862

[2] M. Harif , B. Mallon , C. Patte , et al., “Improving Care for Children With Cancer in Africa: Two Decades of Experience of the French African Pediatric Oncology Group,” JCO Global Oncology 7 (2021): 1509–1512, 10.1200/GO.21.00239. 34678073 PMC8547926

[3] S. C. Howard , A. Davidson , S. Luna‐Fineman , et al., “A Framework to Develop Adapted Treatment Regimens to Manage Pediatric Cancer in Low‐ and Middle‐Income Countries: The Pediatric Oncology in Developing Countries (PODC) Committee of the International Pediatric Oncology Society (SIOP),” Pediatric Blood & Cancer 64, no. S5 (2017): e26879, 10.1002/pbc.26879. 29297619

[4] T. Israels , L. Renner , M. Hendricks , P. Hesseling , S. Howard , and E. Molyneux , “SIOP PODC: Recommendations for Supportive Care of Children With Cancer in a Low‐Income Setting,” Pediatric Blood & Cancer 60, no. 6 (2013): 899–904, 10.1002/pbc.24501. 23441092

[5] B. L. Z. Oh , S. H. R. Lee , and A. E. J. Yeoh , “Curing the Curable: Managing Low‐Risk Acute Lymphoblastic Leukemia in Resource Limited Countries,” Journal of Clinical Medicine 10, no. 20 (2021): 4728, 10.3390/jcm10204728. 34682851 PMC8540602

[6] World Health Organization , “CureAll framework: WHO Global Initiative for Childhood Cancer: Increasing Access, Advancing Quality, Saving Lives,” In: *CureAll Framework: WHO Global Initiative for Childhood Cancer: Increasing Access*, *Advancing Quality*, *Saving Lives*, 2021, https://apps.who.int/iris/handle/10665/347370.

[7] N. Bhakta , L. M. Force , C. Allemani , et al., “Childhood Cancer Burden: A Review of Global Estimates,” Lancet Oncology 20, no. 1 (2019): e42–e53, 10.1016/S1470-2045(18)30761-7. 30614477

[8] S. M. Phillips , L. S. Padgett , W. M. Leisenring , et al., “Survivors of Childhood Cancer in the United States: Prevalence and Burden of Morbidity,” Cancer Epidemiology, Biomarkers & Prevention 24, no. 4 (2015): 653–663, 10.1158/1055-9965.EPI-14-1418. PMC4418452 25834148

[9] T. Lehrnbecher , M. C. Ethier , T. Zaoutis , et al., “International Variations in Infection Supportive Care Practices for Paediatric Patients With Acute Myeloid Leukaemia,” British Journal of Haematology 147, no. 1 (2009): 125–128, 10.1111/j.1365-2141.2009.07844.x. 19663826

[10] E. A. H. Loeffen , L. C. M. Kremer , R. L. Mulder , et al., “The Importance of Evidence‐Based Supportive Care Practice Guidelines in Childhood Cancer—A Plea for Their Development and Implementation,” Supportive Care in Cancer 25, no. 4 (2017): 1121–1125, 10.1007/s00520-016-3501-y. 27928642 PMC5321691

[11] E. A. Loeffen , R. L. Mulder , L. C. Kremer , et al., “Development of Clinical Practice Guidelines for Supportive Care in Childhood Cancer—Prioritization of Topics Using a Delphi Approach,” Supportive Care in Cancer 23, no. 7 (2015): 1987–1995, 10.1007/s00520-014-2559-7. 25516211

[12] J. Seelisch , L. Sung , M. J. Kelly , et al., “Identifying Clinical Practice Guidelines for the Supportive Care of Children With Cancer: A Report From the Children's Oncology Group,” Pediatric Blood & Cancer 66, no. 1 (2019): e27471, 10.1002/pbc.27471. 30259647 PMC6249051

[13] S. Hussain , A. Gray , L. Faughnan , et al., A Global Approach for the Classification and Severity Grading of Essential Adverse Events (EAE) Among Children With Cancer, vol. 67 (Hoboken, NJ: USA: Wiley, 2020), S29.

[14] F. Ceppi , F. Antillon , C. Pacheco , et al., “Supportive Medical Care for Children With Acute Lymphoblastic Leukemia in Low‐ and Middle‐Income Countries,” Expert Review of Hematology 8, no. 5 (2015): 613–626, 10.1586/17474086.2015.1049594. 26013005

[15] C. Rodriguez‐Galindo , P. Friedrich , P. Alcasabas , et al., “Toward the Cure of All Children With Cancer Through Collaborative Efforts: Pediatric Oncology as a Global Challenge,” Journal of Clinical Oncology 33, no. 27 (2015): 3065–3073, 10.1200/JCO.2014.60.6376. 26304881 PMC4979198

[16] S. O. Ekenze , O. C. Okafor , A. A. Obasi , D. C. Okafor , and I. I. Nnabugwu , “Wilms Tumor in Africa: A Systematic Review of Management Challenges and Outcome in Two Decades (2000–2019),” Pediatric Blood & Cancer 67, no. 11 (2020): e28695, 10.1002/pbc.28695. 32909662

[17] A. J. Esbenshade , L. Sung , J. Brackett , et al., “Children's Oncology Group's 2023 Blueprint for Research: Cancer Control and Supportive Care,” Pediatric Blood & Cancer 70, no. S6 (2023): e30568, 10.1002/pbc.30568. 37430431 PMC10528808

[18] L. Andrés‐Jensen , A. Attarbaschi , E. Bardi , et al., “Severe Toxicity Free Survival: Physician‐Derived Definitions of Unacceptable Long‐Term Toxicities Following Acute Lymphocytic Leukaemia,” Lancet Haematol 8, no. 7 (2021): e513–e523. 34171282 10.1016/S2352-3026(21)00136-8

[19] T. Israels , G. M. Afungchwi , L. Klootwijk , et al., “Fever and Neutropenia Outcomes and Areas for Intervention: A Report From SUCCOUR–Supportive Care for Children With Cancer in Africa,” Pediatric Blood & Cancer 68, no. 9 (2021): e29224, 10.1002/pbc.29224. 34245212

[20] R. S. Arora , J. M. Challinor , S. C. Howard , and T. Israels , “Improving Care for Children With Cancer in Low‐ and Middle‐Income Countries—A SIOP PODC Initiative,” Pediatric Blood & Cancer 63, no. 3 (2016): 387–391, 10.1002/pbc.25810. 26797891

[21] A. Suarez , M. Piña , D. X. Nichols‐Vinueza , et al., “A Strategy to Improve Treatment‐Related Mortality and Abandonment of Therapy for Childhood All in a Developing Country Reveals the Impact of Treatment Delays,” Pediatric Blood & Cancer 62, no. 8 (2015): 1395–1402, 10.1002/pbc.25510. 25808195

[22] M. Harif , S. Barsaoui , S. Benchekroun , et al., “Treatment of B‐Cell Lymphoma With LMB Modified Protocols in Africa—Report of the French‐African Pediatric Oncology Group (GFAOP),” Pediatric Blood & Cancer 50, no. 6 (2008): 1138–1142, 10.1002/pbc.21452. 18213709

[23] J. Cayrol , A. Ilbawi , M. Sullivan , and A. Gray , “The Development and Education of a Workforce in Childhood Cancer Services in Low‐ and Middle‐Income Countries: A Scoping Review Protocol,” Systematic Reviews 11, no. 1 (2022): 167, 10.1186/s13643-022-02040-0. 35964146 PMC9375391

[24] L. J. A. Tenniglo , E. A. H. Loeffen , L. C. M. Kremer , et al., “Patients' and Parents' Views Regarding Supportive Care in Childhood Cancer,” Supportive Care in Cancer 25, no. 10 (2017): 3151–3160, 10.1007/s00520-017-3723-7. 28456909 PMC5577054

[25] L. L. Dupuis , S. Cook , P. D. Robinson , D. Tomlinson , E. Vettese , and L. Sung , “Optimizing Symptom Control in Children and Adolescents With Cancer,” Pediatric Research 86, no. 5 (2019): 573–578, 10.1038/s41390-019-0516-3. 31357207

[26] P. D. Robinson , D. Tomlinson , M. Beauchemin , et al., “Identifying Clinical Practice Guidelines for Symptom Control in Pediatric Oncology,” Supportive Care in Cancer 29, no. 11 (2021): 7049–7055, 10.1007/s00520-021-06303-9. 34041614

[27] With Major Processing by Our World in Data . “World Bank Income Groups” [dataset], World Bank, “Income Classifications” [Original Data], Retrieved July 11, 2024 from https://ourworldindata.org/grapher/world‐bank‐income‐groups.

[28] World Health Organization . “WHO Regions,” 2023, https://www.who.int/countries.

